# RNA-binding protein IMP3 is a novel regulator of MEK1/ERK signaling pathway in the progression of colorectal Cancer through the stabilization of MEKK1 mRNA

**DOI:** 10.1186/s13046-021-01994-8

**Published:** 2021-06-21

**Authors:** Meng Zhang, Senlin Zhao, Cong Tan, Yanzi Gu, Xuefeng He, Xiang Du, Dawei Li, Ping Wei

**Affiliations:** 1 Department of Pathology, Fudan University Shanghai Cancer Center, Shanghai, 200032 China; 2 Institute of Pathology, Fudan University, Shanghai, China; 3 Department of Oncology, Shanghai Medical College, Fudan University, Shanghai, China; 4 Department of Colorectal Surgery, Fudan University Shanghai Cancer Center, Shanghai, 200032 China; 5 Biobank, Fudan University Shanghai Cancer Center, Shanghai, China; 6 Cancer Institute, Fudan University Shanghai Cancer Center, Shanghai, China

**Keywords:** IMP3, MEK1/ERK pathway, MEKK1, Colorectal Cancer

## Abstract

**Background:**

MEK1/ERK signaling pathway plays an important role in most tumor progression, including colorectal cancer (CRC), however, MEK1-targeting therapy has little effective in treating CRC patients, indicating there may be a complex mechanism to activate MEK1/ERK signaling pathway except RAS activated mechanism.

**Methods:**

To investigate the clinical significance of IMP3, we analyzed its expression levels in publicly available dataset and samples from Fudan University Shanghai Cancer Center. The effects of IMP3 on proliferation, migration, and invasion were determined by in vitro and in vivo experiments. To investigate the role of IMP3 in colon carcinogenesis, conditional IMP3 knockout C57BL/6 mice was generated. The IMP3/MEKK1/MEK/ERK signaling axis in CRC was screened and validated by RNA-sequencing, RNA immunoprecipitation, luciferase reporter and western blot assays.

**Results:**

We find RNA binding protein IMP3 directly bind to MEKK1 mRNA 3′-UTR, which regulates its stability, promote MEKK1 expression and sequentially activates MEK1/ERK signaling. Functionally, IMP3 promote the malignant biological process of CRC cells via MEKK1/MEK1/ERK signaling pathway both in vitro and in vivo, Moreover, *IMP3*^*−/−*^ mice show decreased the expression of MEKK1 as well as colorectal tumors compared with wild-type mice after treatment with azoxymethane/dextran sodium sulfate. Clinically, the expression of IMP3 and MEKK1 are positive correlated, and concomitant IMP3 and MEKK1 protein levels negatively correlate with metastasis in CRC patients. In addition, MEK1 inhibitor in combination with shRNA-IMP3 have a synergistic effect both in vitro and in vivo.

**Conclusion:**

Our study demonstrates that IMP3 regulates MEKK1 in CRC, thus activating the MEK1/ERK signaling in the progression of colorectal cancer, Furthermore, these results provide new insights into potential applications for combining MEK1 inhibitors with other target therapy such as IMP3 in preclinical trials for CRC patients.

**Supplementary Information:**

The online version contains supplementary material available at 10.1186/s13046-021-01994-8.

## Background

Colorectal cancer (CRC) is one of the leading causes of cancer-related deaths worldwide, and approximately 50% of CRC deaths are due to liver metastasis [1]. At advanced stages, CRC patients often show resistance to traditional treatments. Target therapy has improved overall survival in CRC patients, such as antibodies that targeted epidermal growth factor receptor (EGFR). However, CRC patients with *KRAS* mutations do not respond to these target agents [2]. Activating mutations of *KRAS* are found in around 40% of CRC patients [3], RAS signaling triggers multiple downstream pathways, including RAF-MEK1-ERK1 pathway (also known as MAPK (mitogen activated protein kinase pathway) [4]. As the nature of RAS protein, target of RAS remains challenging, some inhibitors have been invented to target RAF and MEK1, however, there is poorly clinical benefit in monotherapy for the treatment of advanced CRC patients mostly for the feedback reactivation of MEK1/ERK pathway [5]. Other mechanism might exist which need to be further studied.

The activity of MEK1-ERK pathway can be regulated by some RNA-binding proteins (RBPs) [6], RBPs participate in almost the whole life cycle of RNA, including splicing, localization, translation, and decay [7]. With over 1000 RBPs expressed in humans [8], many families of RBPs are involved in the regulation of development, and also contribute to cancer initiation and progression, some prominent RBPs may serve as good therapeutic targets for cancer [9]. To identify RBPs which related with the ability of MEK1-ERK pathway in the progression of CRC, we began by performing high-throughput RNA-sequencing (RNA-seq) of 8 paired CRC with liver metastases samples, including primary tumor tissues(T), normal adjacent tissues(N) and liver metastatic tissues(M).; from this we identified insulin-like growth factor 2 mRNA-binding protein 3 (IMP3) as one of the most dysregulated RBPs in CRC. Through RNA-Seq and RIP-Seq, we noticed that MAPK cascades were enriched in the IMP3 regulatory targets.

IMP3 is a member of the insulin-like growth factor II mRNA-binding protein family [10]. In contrast to IMP1 and IMP2, the biological role of IMP3 has not been well clarified. IMP3 is an oncofetal protein since its re-expression correlates with poor prognosis for tumor patients, and studies on IMP3 have mostly focused on its association with the aggressive behavior of many tumors [11–13]. RNA binding proteins can regulate gene expression at the post-transcriptional level by binding characteristic sequences in the target mRNA [14]. Previous studies showed that IMP3 directly binds to the 3′-UTR of mRNA and regulates its target gene at the post-transcriptional level, with its known targets including CD44 [15], HMGA2 [16], CDK6 [17]. However, the targets and underlying mechanisms of IMP3 relevant to CRC have not yet been elucidated.

Here, we provided evidence that IMP3 directly binds to MEKK1 mRNA 3′-UTR, enhancing its stability and activating the MEK1/ERK pathway. Our study also revealed key functions of IMP3 in the proliferation, migration, and invasion of CRC cells both in vitro and in vivo. In addition, *Villin*-Cre *IMP3*^−/−^ mice were generated and the role of colon-specific IMP3 in colon carcinogenesis was first studied. Clinically, we showed that elevated expression of IMP3 in CRC patients correlated with poor survival, and patients with positive IMP3 and MEKK1 expression had the poorest overall survival (OS) and disease-free survival (DFS). Our results suggest that IMP3 is a novel regulator of /MEK1/ERK signaling pathway through stabilizing MEKK1 mRNA, and suggest that IMP3, combined with MEK1, is a potential therapeutic target for anti-metastatic strategies for CRC.

## Materials

### Clinical samples

A total of 282 patients, who were first diagnosed with colorectal adenocarcinoma at Fudan University Shanghai Cancer Center (FUSCC) during 2008–2009, and who did not undergo any preoperative therapy, were enrolled in this study. The median follow-up time was 81 months, and the longest follow-up time was 97 months. 8 paired primary CRC samples, including primary tumor tissues(T), normal adjacent tissues(N) and liver metastatic tissues(M), were also obtained from CRC patients who were first diagnosed with colorectal adenocarcinoma and liver metastasis at the same time and underwent the surgery of colorectal and liver at the same time at FUSCC during 2018. All patient material was obtained with informed consent, and this study was approved by the institutional review board of Shanghai Cancer Center.

### RNA-seq of clinical samples

RNA-seq was performed using 8 paired primary CRC samples described above, which including adjacent normal adjacent tissues (N), tumor tissues (T), and liver metastatic tissues(M). Total RNA was extracted using the mirVana miRNA Isolation Kit (Ambion) following the manufacturer’s protocol. RNA integrity was evaluated using the Agilent 2100 Bioanalyzer (Agilent Technologies, Santa Clara, CA, USA). The samples with RNA Integrity Number (RIN) ≥ 7 were subjected to the subsequent analysis. The libraries were constructed using TruSeq Stranded Total RNA with Ribo-Zero Gold according to the manufacturer’s instructions. Then these libraries were sequenced on the Illumina sequencing platform (HiSeqTM 2500 or other platform) and 150 bp/125 bp paired-end reads were generated. The differentially expressed mRNAs were defined as a greater than 1.5-fold change between T and N, M and T, with a *p* value < 0.05.

### Cell lines and culture

The colorectal cancer cell lines used in this study, including RKO, HCT116, SW1116, SW480, SW620, HT29, CACO2, and LOVO, were purchased from the Cell Bank of Shanghai Institute of Biochemistry and Cell Biology (Shanghai, China). The cell lines were cultured in DMEM (HyClone, USA) supplement with 10% FBS (Gibco, USA) and 1% complex of penicillin and streptomycin in a 5% CO2 incubator at 37 °C.

### Western blot assays

Antibodies against IMP3, MEKK1, MEK1 were from Abcam, Antibodies against E-cadherin, N-cadherin, MMP2, MMP9, p-ERK1/2, p-MAPK, ERK1/2, p-JNK were from Cell Signaling Technology, all the primary antibodies were used at 1:1000 dilutions. Antibody against GAPDH was from Epitomics, and was used at 1:10000 dilution. All the secondary antibodies were used at 1:5000 dilutions. Western blot assays were performed as previously described [14].

### Quantitative RT-PCR assays

Total RNA from different cells and fresh samples was extracted with Trizol (Invitrogen, USA). cDNA was synthesized using the PrimeScript RT Reagent Kit (Takara, China) following manufacturer’s instructions. Quantitative RT-PCR (qRT-PCR) was performed using SYBR Premix Ex Taq II (Takara, China) according to the manufacturer’s instructions. The relative expression of genes was normalized to GAPDH and calculated by the 2^-ΔΔCT^ method. The specific primers used are listed in supplemental Table 1.

### Plasmid construction and transfections

siRNAs of IMP3 and MEKK1 were purchased from GenePharma (China), the target sequence is IMP3-siRNA1: 5′ GCAGGAAUUGACGCUGUAUTT3’; IMP3-siRNA2: 5′ GCUUCUAUGAAUCUUCAAGTT 3′; MEKK1-siRNA: 5′ CCAUAUAGCCCUGAGGAAATT 3′; Scramble forward: 5′ UUCUCCGAACGUGUCACGUTT 3′.

The human full cDNA sequence of IMP3 and MEKK1 was purchased from GenePharma (China). lentiviruses of IMP3-shRNA and non-target control, lentiviruses of IMP3, MEKK1 and empty vector control were also purchased from GenePharma (China). Transfection and virus infection were performed as previously described [14]. The knockdown or overexpression efficiencies of IMP3 and MEKK1 were confirmed by qRT-PCR and western blot.

### Cell viability assays and colony formation assays

Cells transfected with vectors or siRNAs were re-suspended and seeded into 96-well plates in a density of 5000 cells/well in triplicate. CCK-8 reagent (10 μl, Dojindo, Japan) was added to each well at 0, 24, 48, 72 and 96 h. The absorbance at 450 nm was measured after 2 h incubation at 37 °C. For the colony formation assay, 500 to 1000 cells were seeded into a 6-well plate and cultured in a media containing 10% FBS for 14 days. The colonies were then fixed and stained, and the number of colonies counted.

### Cell migration and invasion assays

The cell migration ability was accessed by wound-healing assay. Linear wound was generated with a 200 μl pipette tip until cell confluence. Wound closure was examined and photographed at pre-determined time points (0, 24 h) in multiple microscopic regions.

And the cell invasion ability was accessed by the Transwell assay using 24-Well Cell Invasion Assay (Corning, NY, USA) with Matrigel (BD Biosciences, NJ, USA). 24 h after transfection, 3 × 10^4^ cells with FBS-free medium were seeded into the upper chamber, and medium containing 20% FBS was applied to the lower chamber as chemoattractant. After 48 h incubation, cells attached to the lower surface of the chamber were fixed with ethanol, stained with 0.5% crystal violet, and then photographed.

### Animal studies

Animal experiments were approved by the Ethics Committee at FUSCC. Briefly, male BALB/c nude (Shanghai Slac Laboratory Animal Co. Ltd., 4–6 weeks) were subcutaneously injected with IMP3 stable knockdown HCT116 cells (2 × 10^6^ suspended in 0.1 ml PBS for each mouse) and IMP3-overexpressing LOVO cells (2 × 10^6^ suspended in 0.1 ml PBS for each mouse). For the subcutaneous xenograft model, tumor growth with a digital caliper was measured every 3 days. Approximately one month after implantation, the mice were killed and the tumors were removed. Tumor volume was calculated as (width)^2^ × length/2.Once reaching an average tumor volume of 100 mm^3^,

They were intraperitoneally treated with u0126 (20 mg/kg). Administration of vehicle or agents and measurement of tumor volume were done every 3 days. Finally, mice were weighed and sacrificed, and tumors were weighed and dissected. IHC of xenograft tumors were done according to the protocol above. For in vivo metastasis assays, 5 × 10^6^ cells were injected into the tail vein; 6 weeks later, all mice were killed and the lungs removed for pathological examination and hematoxylin and eosin (H&E) staining.

### Luciferase reporter assays

Luciferase reporter assay was performed as previously described [14]. The MEKK1–3′-UTR sequence was cloned into a luciferase reporter gene vector pGL3 basic vector as pGL3-MEKK1 3′-UTR. Site-specific mutagenesis of the MEKK1–3′-UTR was carried out using a QuikChange Site-Directed Mutagenesis kit (Stratagene, La Jolla, CA, USA), according to the manufacturer’s instructions. The indicated promoters were transfected into HEK-293 T and CRC cells. Twenty-four hours after transfection, the cells were harvested and then assayed for luciferase activity with the Dual-Luciferase Reporter Assay System (Promega, Madison, WI, USA). The promoter activity was normalized by co transfection with Renilla luciferase reporter.

### RNA immunoprecipitation (RIP) assays

The Magna RIP Kit (17–701; EMD Millipore, Billerica, MA, USA) was used for the RIP assay in RKO cells with high IMP3 expression, according to the manufacturer’s protocol. Briefly, RKO cells were grown in four 15-cm dishes for three days and then washed three times with ice-cold PBS and centrifugation to collect the cells. The cells were collected and resuspended in 200.

μL of RIP buffer and then incubated with magnetic protein A/G beads conjugated with the indicated antibodies at 4 °C for 3 h. A 100-μL aliquot of the supernatant was diluted with 900 μL of RIP buffer and then treated with proteinase K solution to isolate IMP3 protein-associated RNA from the eluted immunocomplexes. RNA was then isolated and extracted by using the phenol/chloroform method for RIP-sequencing (RIP-seq), and the level of MEKK1 mRNA in the RIP complex was assayed by using qRT-PCR.

### RNA-seq and data analyses

Stable IMP3 knockdown and overexpressed CRC cells were subjected to RNA-seq to detect the differentially expressed mRNAs regulated by IMP3. Briefly, total RNA was isolated from these cells using the phenol/chloroform method and then subjected to RNA-seq. The sequencing protocol is the same as the clinical samples. The differential genes were selected using a fold change > 1.5, and Gene Ontology (GO) and Kyoto Encyclopedia of Genes and Genomes (KEGG) pathway enrichment analyses were used for pathway enrichment using DAVID (https://david.ncifcrf.gov/) with a significance threshold of *p* < 0.01.

### Immunohistochemistry (IHC)

The protein expression levels of IMP3, MEKK1, MEK1 and p-ERK were determined by IHC analysis using CRC tissue arrays constructed previously [18]. Briefly, the slides were incubated with primary antibodies: IMP3 (EPR12021, 1:100, Abcam, USA); MEKK1 (2F6, 1:100, Abcam, USA); MEK1 (Y77, 1:100, Abcam, USA); p-ERK (ab79483, 1:100, Cell Signaling Technology, USA), at 4 °C overnight. The subsequent steps were performed using the EnVision Detection System (Dako, USA).

### Generation of conditional IMP3 knockout C57BL/6 mice

We constructed a double-LoxP IMP3-targeting vector to generate a ‘fl’ mouse IMP3-targeted locus consisting of a LoxP site inserted into the IMP3 exon and the other LoxP site inserted in the 3′-UTR of IMP3. The colon-specific *Villin* promoter–driven Cre Recombinase (Villin-Cre) TG C57BL/6 mice were purchased from Jackson Laboratories, Bar Harbor, ME. The *Villin*-Cre TG C57BL/6 mice were bred with *IMP3* fl/fl C57BL/6 mice and the offspring were screened by polymerase chain reaction (PCR) for *Villin*-Cre IMP3 fl/+ mice. These mice then were back-crossed with *IMP3* fl/fl C57BL/6(*IMP3* ^fl/fl^) mice to generate *Villin*-Cre *IMP3* fl/fl C57BL/6 (*IMP3*^−/−^) mice. *IMP3*^−/−^ mice were bred with *IMP3* fl/fl C57BL/6 mice to generate a sufficient number of mice for the colorectal tumor experiments. *IMP3* floxed allele or recombined null allele mice were detected by PCR amplification using Primer1(*IMP3*) forward: 5′ GACCACAACCTCCACGAACT 3′, reverse: 5′ AGTCCACCCAGGTGACAAAG 3′; Primer2(*Cre*) forward:5′ GTGTGGGACAGAGAACAAACC3’, reverse:5′ ACATCTTCAGGTTCTGCGGG 3′. These sets of primers amplify a 666–base pair band in the floxed allele and a 415-base pair band in the null allele, and 1100-base pair band in the *Villin*-cre allele.

### AOM/DSS-induced colitis-associated cancer model

*IMP3*^fl/fl^ and *IMP3*^−/−^ mice were injected intraperitoneally with azoxymethane (AOM, Sigma-Aldrich, USA) at a dose of 7 mg/kg body weight. After 7 days, mice were given 3 cycles of 3% dextran sodium sulfate (DSS) for 7 days in sterile drinking water, followed by 14 days of regular sterile drinking water. Loss of body weight in these mice was monitored daily, and mice with > 20% body weight loss were considered to have reached a humane end-point and killed. After completion of the entire AOM/DSS regimen, these mice were sacrificed (at day 86), and their colons removed and cut longitudinally. The number and sizes of tumors in the colon of each mouse were analyzed and measured. Then pathological examination and hematoxylin and eosin (H&E) staining was performed on the colons.

### Statistical analysis

Experiments were independently repeated at least 3 times, and results are presented as mean ± SD, and for the figures, the representative images were showed. Differences between groups were calculated by the Student’s t test and the χ^2^ test. Kaplan-Meier analysis and log-rank tests were applied to determine differences in survival. Cox univariate and multivariate proportional hazards regression models were used to determine the independent factors influencing survival. All statistical analyses were carried out using SPSS 20.0 (IBM, New York). *p*values < 0.05 were considered as statistically significant.

## Results

### Elevated IMP3 expression correlates with poor prognosis in CRC patients

**Fig. 1:**
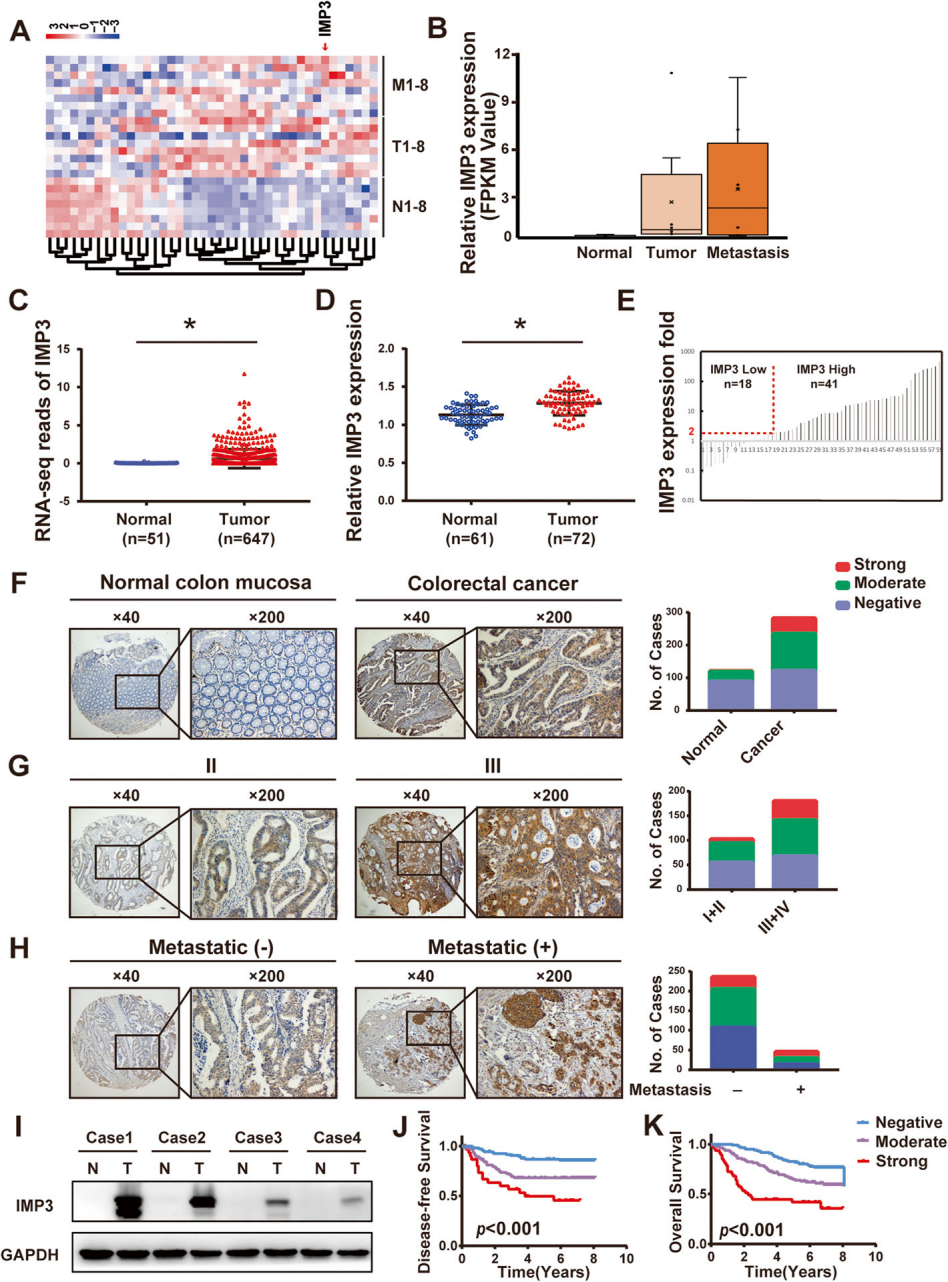
IMP3 is upregulated in human CRC patients and predicted poor prognosis. (**A**) Heatmap of differentially expressed mRNAs related to RNA binding proteins between adjacent normal mucosa (N), cancer tissue (T), and liver metastases tissue (M) from 8 paired colon cancer tissues. (**B**) IMP3 mRNA expression level in adjacent normal mucosa (N), cancer tissue (T), and liver metastases tissue (M) from 8 paired colon cancer tissues. (**C**) Expression of IMP3 in the TCGA CRC RNAseq dataset (Normal *n* = 51, Tumor *n* = 647). (**D**) Expression of IMP3 in the FUSCC dataset (Normal *n* = 61, Tumor *n* = 72). (**E**) IMP3 expression in 59 paired CRC tissues and its adjacent normal mucosa. (**F-H**) The expression of IMP3 in CRC tissue microarrays by IHC. (**F**) IMP3 was overexpressed in CRC tissues (*p* < 0.05). (**G**) IMP3 overexpression was associated with AJCC stage (*p* < 0.05). (**H**) MP3 overexpression was associated with tumor distal metastasis (*p* < 0.05). (**I**) Representative images of IMP3 expression in CRC tissues by Western blot analysis. (**J**) High IMP3 expression predicted shorter DFS than that of patients with low IMP3 expression. (**K**) High IMP3 expression predicted shorter OS than that of patients with low IMP3 expression

To identify key RNA binding proteins involved in the progression of CRC, we began by performing high-throughput RNA-Seq analysis of 8 paired primary CRC and liver metastases samples, including primary tumor tissues(T), normal adjacent tissues(N) and liver metastatic tissues(M). We then identified overlapping RBP genes that were different between all 3 tissue types, finding a total of 41 RBP genes (Fig. 1A). In addition, when using cut-off points of a fold change > 2 and *p* < 0.05, only IMP3 was found to increase from N to T tissue, and increase again from T to M tissue (Fig. 1B).

To validate the increased expression level of IMP3 in CRC, we next investigated the level of IMP3 in CRC tissues from the publicly available ‘The Cancer Genome Atlas’ (TCGA) dataset. Data showed that IMP3 was significantly upregulated in colorectal tumor tissues (*n* = 647) when compared with normal epithelial tissues (*n* = 51, *p* < 0.05, Fig. 1C). In the FUSCC dataset, the mRNA levels of IMP3 in CRC tissues (*n* = 72) were also higher than the levels in normal mucosa (*n* = 61, *p* < 0.05, Fig. 1D). Furthermore, from 59 paired CRC and normal tissues, IMP3 mRNA levels were at least twofold higher in cancerous tissue than in normal tissue in nearly 70% of pairs (41/59, Fig. 1E). IHC and western blot analysis found that IMP3 protein levels were higher in CRC tissues compared to normal mucosa tissues (Fig. 1F, I, Table 1). IMP3 overexpression was associated with tumor stage and metastasis (Fig. 1G, H, *p* < 0.05). Kaplan-Meier curves showed that patients with higher expression of IMP3 had poorer DFS and OS than those with relatively low IMP3 expression (Fig. 1J, K).

**Table 1:** Expression of IMP3, MEKK1, MEK1 and p-ERK in 114 cases of colorectal cancer and adjacent normal mucosa tissues.

Markers	CRC tissues	Adjacent normal mucosa tissues	*P* value
IMP3			
Positive	71	30	<0.001*
Negative	43	84	
MEKK1			
Positive	84	37	<0.001*
Negative	30	77	
MEK1			
Positive	73	32	<0.001*
Negative	41	82	
p-ERK			
Positive	81	31	<0.001*
Negative	33	83	

### Colon-specific conditional *IMP3* knockout mice showed decreased AOM/DSS treatment-induced colon carcinogenesis

**Fig. 2:**
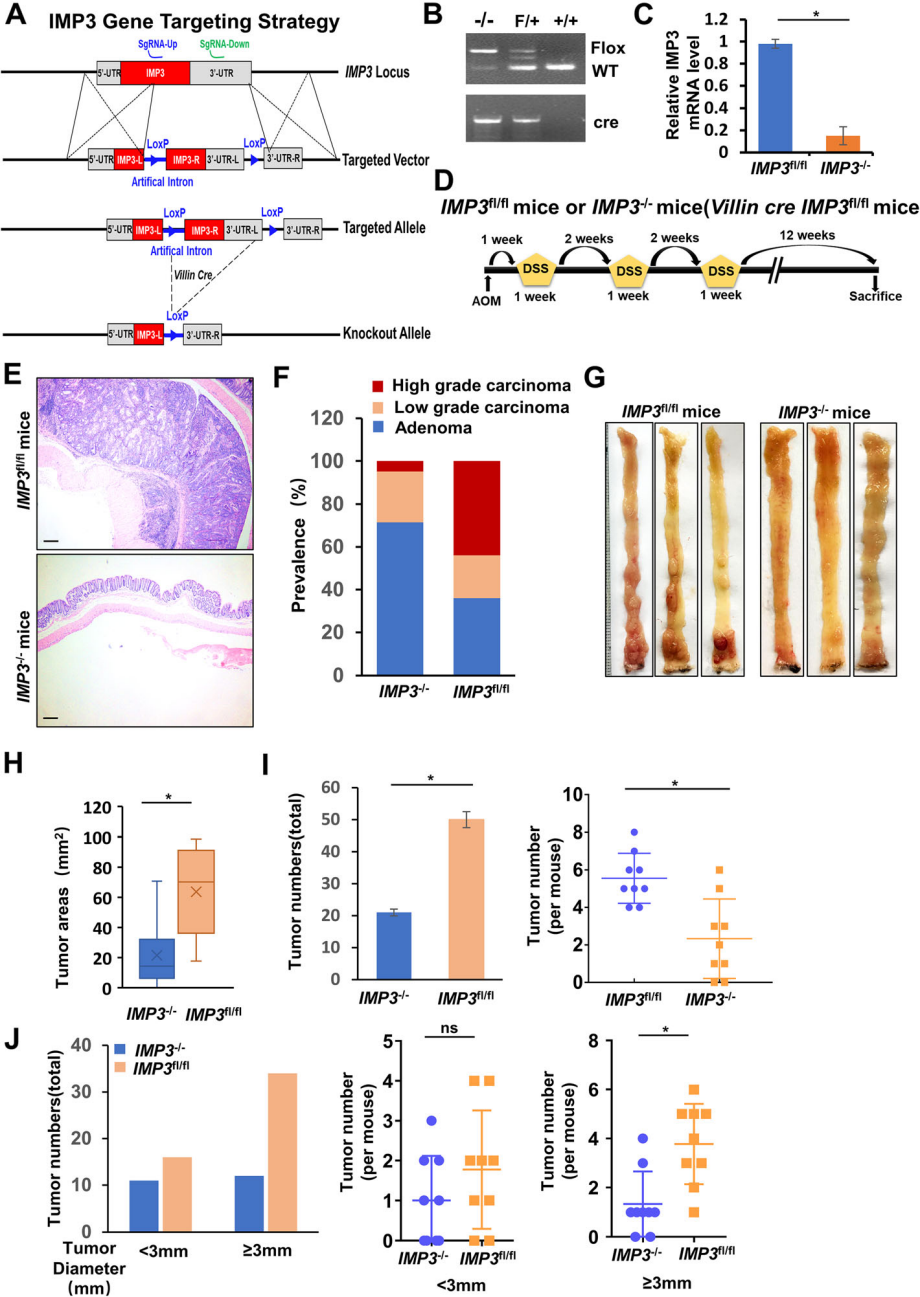
Colon-specific knockout of IMP3 decreased AOM/DSS induced colon carcinogenesis. (**A**) Scheme of *Villin*-*Cre*-mediated recombination of *IMP3* floxed allele. (**B**) PCR amplification of genomic DNA. *Villin*-*Cre IMP3*^−/−^ colon shows a 666 base pair band specific to recombination allele. (**C**) The quantitative real-time RT-PCR analysis of *Villin*-*Cre IMP3*^−/−^colon tissues shows an approximately 90% decrease of IMP3 mRNA levels compared with the *IMP3*^fl/fl^ colon tissues. (**D**) Six- to 8-week-old *IMP3*^fl/fl^ (*n* = 9) and *Villin*-*Cre IMP3*^−/−^mice (*n* = 9) were subjected to a single intraperitoneal injection of AOM (10 mg/kg/body weight) followed by 3 cycles of 1 week of administration of 2.5% DSS in the drinking water, each cycle separated by a 2-week period. Mice were killed at 12 weeks after AOM/DSS exposure. (**E**) HE staining of representative colon tissue image of *Villin*-*Cre IMP3*^−/−^ and *IMP3*^fl/fl^ mice. (**F**) Prevalence of low-grade adenomas, highgrade adenomas, and adenocarcinomas of *Villin*-*Cre IMP3*^−/−^ and *IMP3*^fl/fl^ mice. (**G**) Macroscopic finding of *Villin*-*Cre IMP3*^−/−^ and *IMP3*^fl/fl^ mouse colons. (**H**)Tumor areas, *Villin*-*Cre IMP3*^−/−^ mice showed a significant reduction in the total tumor areas compared with *IMP3*^fl/fl^ mice. (**I**) Tumor numbers, *Villin*-*Cre IMP3*^−/−^ mice showed a significant reduction in the total tumor numbers(left) and per mouse tumor number(right) compared with *IMP3*^fl/fl^ mice. (**J**) *Villin*-*Cre IMP3*^−/−^ mouse colons showed a decreased total tumor numbers or per mouse tumor numbers in which the diameter was larger than 3.0 mm, while no significant difference was found in tumor diameter was smaller than 3.0 mm

To investigate the role of IMP3 in colon carcinogenesis, we used the colon-specific *Villin*-Cre recombinase transgene (*Villin*-Cre) to conditionally delete the *IMP3* floxed targeted allele (Fig. 2A). We identified the presence of the *IMP3* floxed allele using specific PCR primers, these resulted in an *IMP3* floxed allele band of approximately 666 bp, while the wild-type band was approximately 415 bp (Fig. 2B). qRT-PCR analysis showed decreased *IMP3* mRNA levels in colon tissues of *IMP3*^−/−^ (*Villin*-Cre *IMP3*^fl/fl^) mice compared with *IMP3*^fl/fl^ mice. (Fig. 2C).

To induce colon cancer, 9 *IMP3*^fl/fl^ and 9 *IMP3*^−/−^ mice were subjected to a single intraperitoneal injection of AOM (10 mg/kg body weight), followed by 3 cycles of a 1-week application of 2.5% DSS to the drinking water; each of these cycles was separated by a 2-week interval (Fig. 2D). After 12 weeks of AOM/DSS exposure, all the *IMP3*^fl/fl^ mouse colons had developed adenocarcinomas. Mouse colons were dissected and examined for tumors under the dissecting microscope and then fixed and paraffin-embedded. Histological examination of H&E-stained sections found that among the mice bearing colon tumors, the percentages of adenomas, low-grade adenocarcinomas and high-grade adenocarcinomas in AOM/DSS-administered *IMP3*^fl/fl^ mice were 36%(18/50), 20%(10/50), and 44%(22/50), respectively, whereas the corresponding percentages in AOM/DSS-administered *IMP3*^−/−^ mice were 71.43%(15/21), 23.81%(5/21) and 4.76(1/21)%, respectively (Fig. 2E,F,G). It is interesting to note that while only one high-grade adenocarcinoma was observed from among all of the IMP3.

^−/−^ mice, this tumor type accounted for nearly half of all the tumors found in *IMP3*^fl/fl^ mice. Colon lengths were similar in AOM/DSS-administered *IMP3*^fl/fl^ and *IMP3*^−/−^ mice (Fig. 2G). *IMP3*^−/−^ mice showed a statistically significant reduction in the total tumor area and in the number of colon tumors compared with *IMP3*^fl/fl^ mice (Fig. 2H, I). Quantitatively, *IMP3*^−/−^ mice showed a statistically significant reduction in the number of tumors ≥3 mm in diameter, but no significant reduction in the number of tumors < 3 mm in diameter (Fig. 2J, K). These results indicated that colon-specific IMP3 knockout decreased AOM/DSS-induced colon tumorigenesis and tumor growth.

### IMP3 promotes the proliferation, migration, and invasion of CRC cells in vitro

**Fig. 3:**
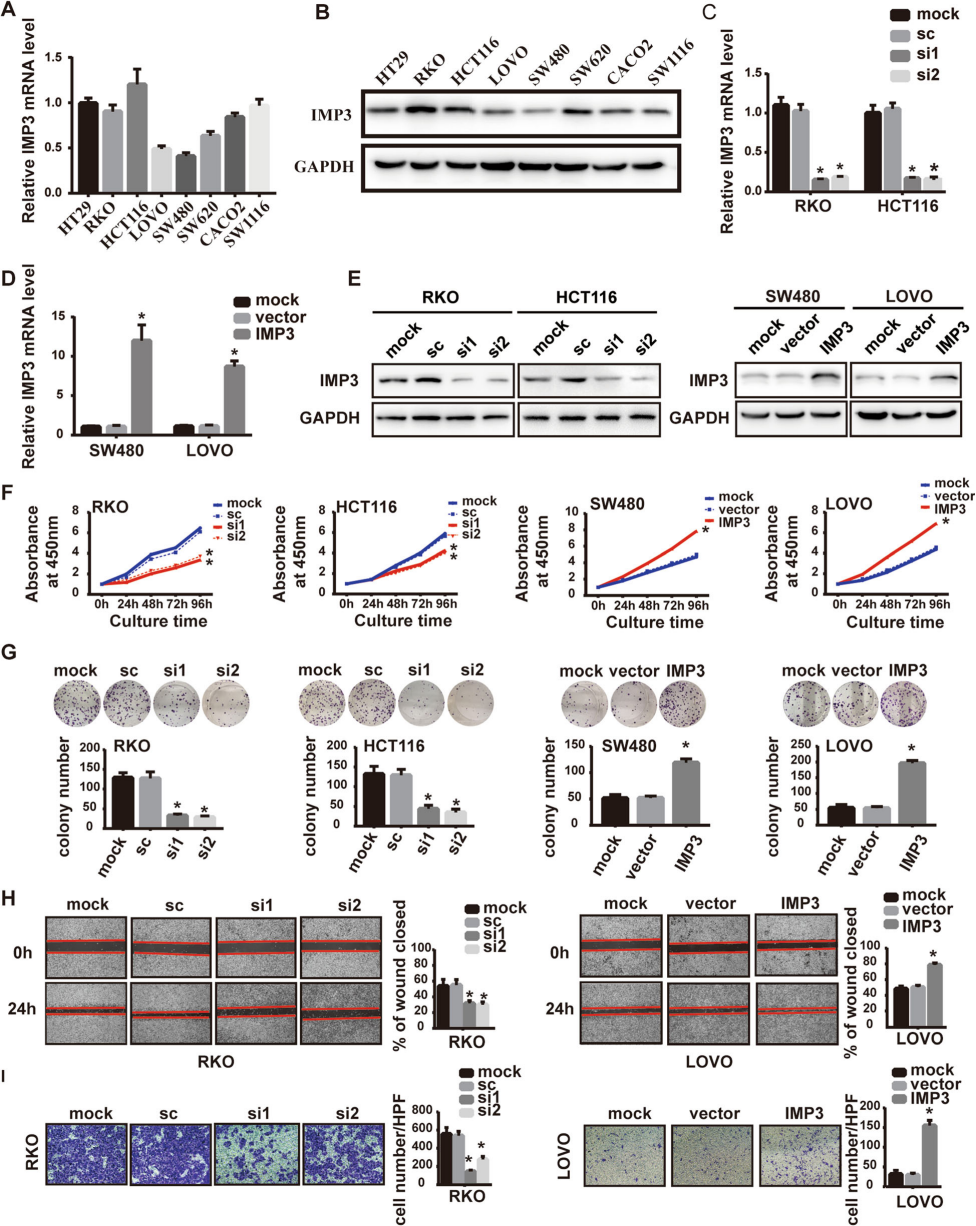
IMP3 promoted CRC cell proliferation, migration and invasion in vitro. (**A**) The baseline RNA level of IMP3 in eight CRC cell lines detected by RT-qPCR. (**B**) The baseline protein level of IMP3 in eight CRC cell lines detected by western blot. (**C**) Efficiencies of IMP3 knockdown was detected in indicated cells transfected with siRNAs by RT-qPCR (* *p* < 0.05). (**D**) Efficiencies of IMP3 overexpression was detected in indicated cells transfected with plasmids by RT-qPCR (* *p* < 0.05). (**E**) Efficiencies of IMP3 knockdown or overexpression was detected in indicated cells transfected with siRNAs or plasmids by western blot. (**F**) CCK8 assays showed that knockdown of IMP3 reduced cell viabilty in RKO and HCT116 cell line (* *p* < 0.05), and upregulation of IMP3 increased cell viability in LOVO and SW480 cell line (* *p* < 0.05). (**G**) Colony formation assays for indicated cells after transfection with siRNAs or plasmids (* *p* < 0.05). (**H**) Representative images (40×) of wound healing assays for indicated cells (* *p* < 0.05). (**I**) Representative images (200×) of transwell invasion assay for indicated cells (* *p* < 0.05)

To identify the role of IMP3 in CRC cells, we first examined the baseline expression of IMP3 in eight CRC cell lines (HT29, RKO, HCT116, LOVO, SW480, SW620, CACO2, and SW1116). Results showed that IMP3 was relatively higher in RKO and HCT116 cells, and lower in LOVO and SW480 cells (Fig. 3A, B). Next, RKO and HCT116 cells were transfected with the siRNAs specific to IMP3, while LOVO and SW480 cells were transfected with IMP3 full-length plasmids. Transfection efficiencies were quantified by qRT-PCR and western blot assays (Fig. 3C-E). CCK-8 assays indicated that IMP3 knockdown significantly reduced CRC cell proliferation. Similarly, the colony formation assays demonstrated that IMP3 knockdown decreased the number of colonies formed over 14 days (Fig. 3F, G). In contrast, forced expression had the opposite effect in LOVO and SW480 cells, when tested by either the CCK-8 assay or the colony formation assay (Fig. 3F, G). We further analyzed the role of IMP3 expression in CRC cell metastasis. The wound healing assays showed that cells with higher IMP3 expression exhibited stronger flattening and spreading abilities (Fig. 3H, Supplementary Fig. 1A, B). This result was also confirmed by transwell assays, cells with altered IMP3 expression levels possessed different invasive abilities (Fig. 3I, Supplementary Fig. 1C, D). Collectively, these data suggest that IMP3 promotes the proliferation, migration, and invasion of CRC cells in vitro.

### IMP3 directly binds to MEKK1 and regulates the MEK1/ERK pathway

**Fig. 4:**
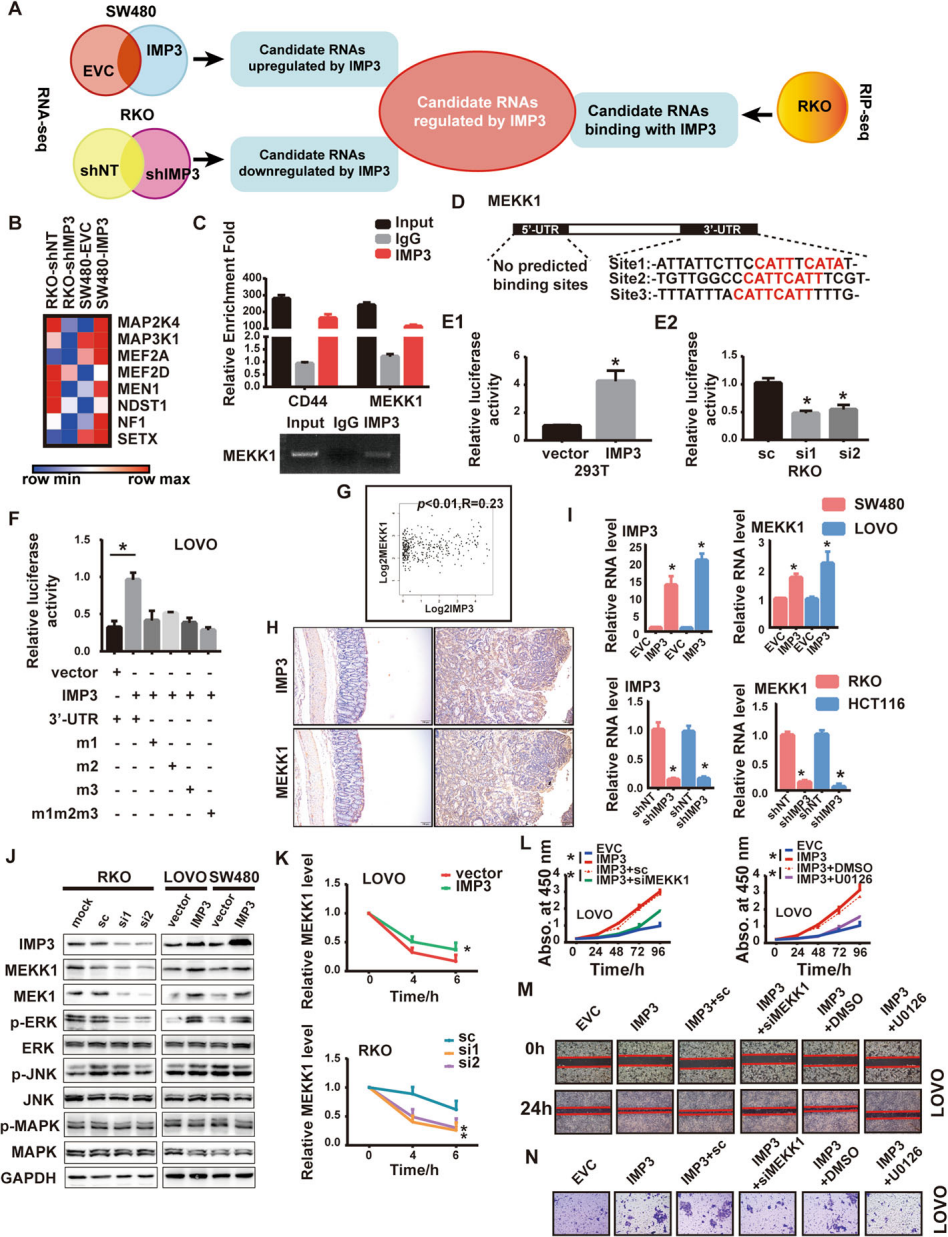
IMP3 directly binds to MEKK1 and activates MEK/ERK pathway. (**A**) Flow chart of bioinformatics analysis predicting candidate RNAs regulated by IMP3. (**B**) MAPK cascades were enriched in IMP3 regulated targets. (**C**) RIP assay showing that MEKK1 interacted with IMP3 in RKO cells. The RT-qPCR products were analyzed by electrophoresis (below) (*p < 0.05), CD44 was used as a positive control. (**D**) The schematic diagram of IMP3 potential binding site in MEKK1 UTR area. E-F. Luciferase reporter assay, (**E1**)293T cell was transfected with IMP3, (E2) RKO cell was transfected with IMP3 siRNAs, (**F**)LOVO cell was co-transfected with IMP3 and MEKK1 3’UTR wt and indicated mutations. The luciferase assay data were normalized to firefly luciferase activity. (*p < 0.05). (**G**)GEPIA analyzed the expression of IMP3 and MEKK1 in CRC tissues. (**H**) Representative images of IHC staining of IMP3 and MEKK1 in the colon tissues from *Villin*-*Cre IMP3*^−/−^ and *IMP3*^fl/fl^ mice treated with AOM/DSS. (**I**) The mRNA level of MEKK1 in indicated cells (* *p* < 0.05). (**J**) The protein level of MEKK1 and its downstream pathways in indicated cells. MEK1: mitogen-activated protein kinase kinase 1; ERK: extracellular-signal-regulated kinase; JNK: c-Jun N-terminal kinase; MAPK: mitogen-activated protein kinase; The prefix p represents its phosphorylation. (**K**) RT-qPCR analysis of MEKK1 mRNA stability in actinomycin D treated CRC cells (* *p* < 0.05). (**L**) CCK-8 results showed that knockdown of MEKK1 or using MEK/ERK inhibitor U0126 attenuated the enhanced cell viability induced by overexpression of IMP3 in LOVO cells (* *p* < 0.05). Representative images of (**M**) wound healing assays and (**N**) transwell assays showed that MEKK1 repression or U0126 rescued the enhanced invasion and migration ability by overexpression of IMP3 (* *p* < 0.05)

To elucidate the underlying mechanism of underlying the role of IMP3 in CRC, we analyzed candidate RNAs regulated by IMP3 in IMP3-knockdown, IMP3-upregulated and relative control CRC cells (RKO and SW480 cell lines) using high-throughput RNA-sequencing (RNA-seq), and characterized the candidate RNAs binding with IMP3 using RIP-seq in the RKO cell lines. Analyzing these results together, we focused on differentially expressed transcripts that were reproducibly associated with IMP3 in RIP-seq assays, and found 622 transcripts (Fig. 4A). Interestingly, we noticed that MAPK cascades were enriched in the IMP3 regulatory targets (Fig. 4B and Supplemental Fig. 2). Moreover, the RIP assay confirmed the interaction between MEKK1(also known as MAP3K1) and IMP3 in extracts from RKO cells. CD44 was used as the positive control, which was a known target [15] (Fig. 4C). Collectively, these results demonstrated a direct interaction between MEKK1 and IMP3.

To investigate whether MEKK1 is a direct target of IMP3 in CRC, bioinformatics analysis was performed to identify the potential binding site. Firstly, we found the potential IMP3 binding site sequence (http://cisbp-rna.ccbr.utoronto.ca/). As RBPs participate in posttranscriptional regulation usually via the 3′-UTR and 5′-UTR, we compared the sequence of MEKK1 mRNA 3′-UTR and 5′-UTR with the IMP3 potential binding site. Results showed that the MEKK1 3′-UTR contains multiple potential IMP3 binding sites, while there is no potential binding site in the 5′-UTR (Fig. 4D). Luciferase activity was enhanced by overexpressed IMP3 in 293 T and decreased by knockdown IMP3 in RKO cells (Fig. 4E1&2). These results suggested that IMP3 regulates MEKK1 via direct binding to its 3′-UTR.To further confirm this result, we next used a luciferase reporter gene vector to generate recombinant plasmids containing mutated binding site sequences. Compare with the wild-type group, the luciferase intensity was not enhanced by IMP3 with the mutant 3′-UTR plasmids versus the WT 3′-UTR plasmid (Fig. 4F). In concordance with these results, bioinformatics analysis indicated that the expression of MEKK1 was related to IMP3 expression in CRC tissues (*p* < 0.01, *r* = 0.23, Fig. 4G, GEPIA: http://gepia.cancer-pku.cn/). qRT-PCR and western blot assays revealed that altered IMP3 expression in CRC cell lines positively regulated MEKK1 mRNA and protein levels (Fig. 4I, J). Moreover, IHC of transgenic mice tumor tissue also showed that IMP3 expression level is positively correlated with MEKK1 expression level (Fig. 4H). Three classical pathways lie downstream of MEKK1, the MEK1/ERK pathway, the JNK pathway, and the MAPK pathway. Western blot assays showed that IMP3 upregulated MEKK1 and then activated the MEK1/ERK pathway, whereas it only exhibited a slight effect on the JNK and MAPK pathways (Fig. 4J). Next, to confirm whether IMP3 could affect the stability of MEKK1 mRNA, we used actinomycin D to inhibit transcription and qRT-PCR was performed to measure the decay rate of MEKK1. As expected, we confirmed that MEKK1 was more stable in cells overexpressing IMP3 (Fig. 4K). Thus, IMP3 promotes the MEK1/ERK pathway by directly interacting with MEKK1 and retarding its mRNA decay.

To further confirm whether IMP3 promotes CRC progression through the MEKK1/MEK1/ERK pathway, we performed a series of functional restoration assays using either an siRNA or plasmid for MEKK1, or the MEK1/ERK signaling specific inhibitor U0126, in CRC cell lines. U0126 was used at a concentration 10Um, as this was the IC50 according to our CCK-8 assays (Supplementary Fig. 3A, B). The results from these experiments showed that knockdown of MEKK1 or using U0126 reduced the proliferation of IMP3 overexpression cells (Fig. 4L, Supplementary Fig. 3D, E. In addition, overexpression of MEKK1 increased cell proliferation in IMP3-knockdown cells (Supplementary Fig. 3C). Similarly, the effect of IMP3 on the invasion and migration of CRC cells was also attenuated by knockdown of MEKK1 or using U0126 (Fig. 4M, N, Supplementary Fig. 3F-O). Therefore, these results showed that the MEKK1/MEK1/ERK pathway participates in IMP3-facilitated CRC cell proliferation, migration, and invasion.

Moreover, to identify the role of MEKK1 in CRC, a series of functional assays were performed. CCK-8 assays indicated that MEKK1 knockdown significantly reduced CRC cell proliferation (Supplementary Fig. 4A). Similarly, forced expression of MEKK1 had the opposite effect in LOVO cells (Supplementary Fig. 4A). Wound healing assays showed that cells with lower MEKK1 expression exhibited weaker flattening and spreading abilities (Supplementary Fig. 4B). This result was also confirmed by transwell assays, cells with altered MEKK1expression levels possessed different invasive abilities (Supplementary Fig. 4C). These results suggest that MEKK1 has the similar function of IMP3 in CRC.

### IMP3 promotes the progression of CRC, and sensitizes CRC cell responses to the MEK1 inhibitor U0126 in vivo

**Fig. 5:**
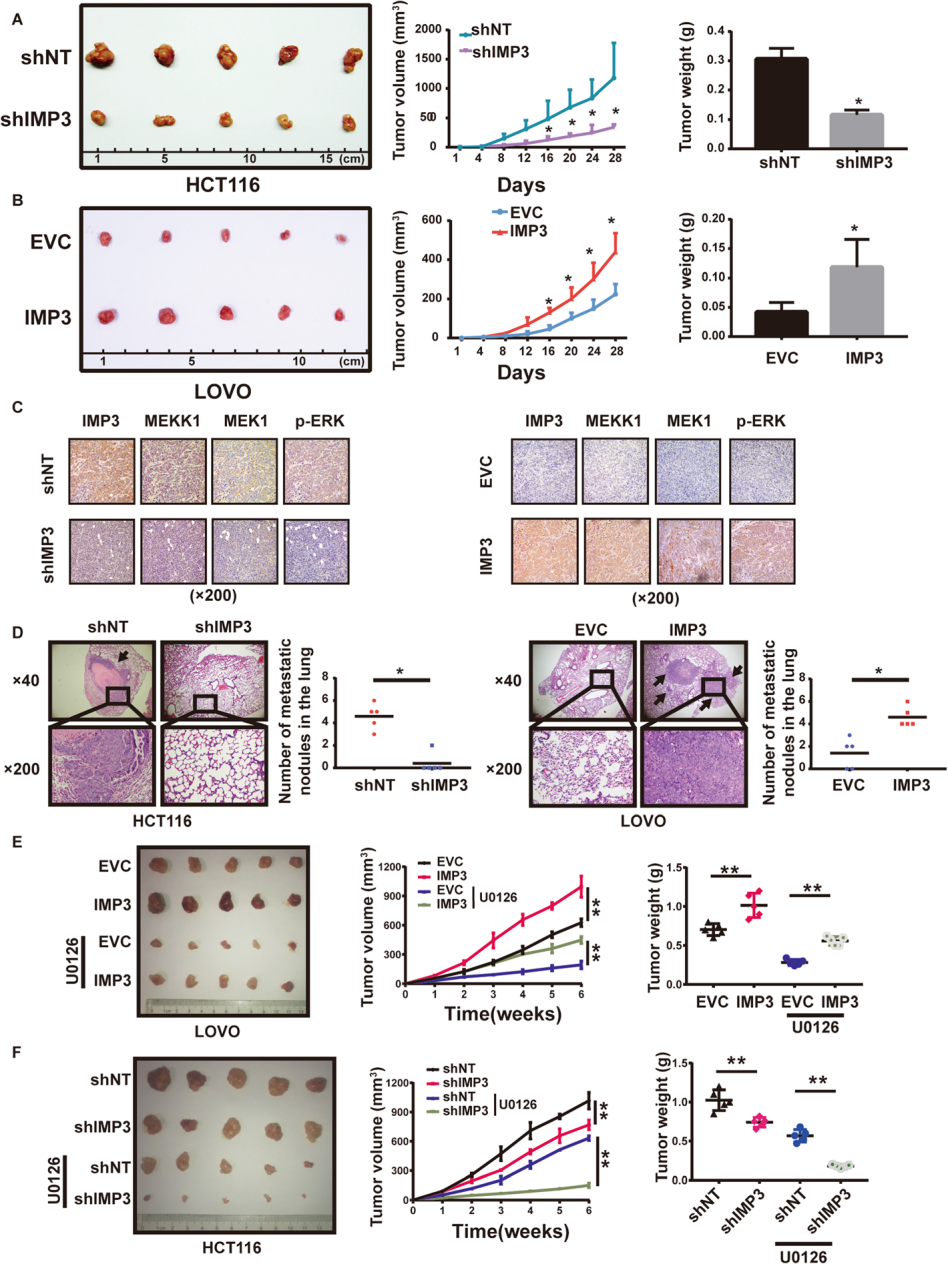
IMP3 promotes CRC cell proliferation and metastasis in vivo. (**A-B**) 2 × 10^6^ stable cells were injected subcutaneously into the groin of nude mice (*n* = 5 for either group). (**A**) Knockdown of IMP3 declined tumor growth and tumor weights compared with those control cells in HCT116 (**B**) Overexpression of IMP3 promoted tumor growth and tumor weights compared with those control cells in LOVO. (**C**) Representative images of IHC staining for IMP3, MEKK1, MEK1 and p-ERK. (**D**) Representative images of lung metastasis in nude mice with HE staining. (left) Knockdown of IMP3 reduced the number of lung metastasis nodular compared with those control cells in HCT116, (right) Overexpression of IMP3increased the number of lung metastasis nodular compared with those control cells in LOVO (* *p* < 0.05). (**E**) Representative image of nude mice bearing tumors formed by overexpression of IMP3 in LOVO and their control cells after U0126 treatment. The average tumor volume and tumor weight of nude mice bearing tumors formed by overexpression of IMP3 in LOVO and their control cells after U0126 treatment. (* *p* < 0.05, ** *p* < 0.05) (**F**) Representative image of nude mice bearing tumors formed by stable knockdown of IMP3 in HCT116 and their control cells after U0126 treatment. The average tumor volume and tumor weight of nude mice bearing tumors formed by stable knockdown of IMP3 in HCT116 and their control cells after U0126 treatment. (* *p* < 0.05, ** *p* < 0.05)

To explore the in vivo effect of IMP3 on CRC cell proliferation, stable cells with IMP3 overexpression or knockdown were injected subcutaneously into nude mice. Overexpression of IMP3 quickened tumor growth and increased overall tumor weights, while knockdown of IMP3 had the opposite effect (Fig. 5A&B). IHC of xenograft tissues showed that overexpression of IMP3 promoted the expression of MEKK1, MEK1 and p-ERK, while knockdown of IMP3 had the opposite effects (Fig. 5C). We next evaluated the in vivo effect of IMP3 on metastasis. Six weeks after the indicated cells were injected into the tail vein of nude mice, the number of nodules in the lung was greater in groups with higher IMP3 expression, than in groups with relatively lower IMP3 expression (Fig. 5D). Consistent with the in vitro results obtained in CRC cell lines, IMP3 could promotes the tumorigenesis and metastasis of CRC cells in vivo.

In addition, we examined whether the MEK1 inhibitor U0126 could affect the biological processes by which IMP3 promotes tumorigenesis, or whether modified IMP3 expression could affect the sensitivity of CRC cell lines to U0126 in vivo. Stable cells with modified IMP3 expression were injected subcutaneously into nude mice, and when the tumor volume had reached approximately 100 mm^3^, mice were treated with U0126 on alternate days. The results showed U0126 could partially reverse the effects of IMP3 on tumorigenesis (Fig. 5E), and knockdown of IMP3 concomitant with U0126 treatment could further reduce tumor volumes and weights in vivo (Fig. 5F). These data confirmed that IMP3 activated MEKK1/MEK1/ERK signaling in vivo.

### Clinical association between IMP3 and MEKK1, and MEK1/ERK signaling in CRC tissues

**Fig. 6:**
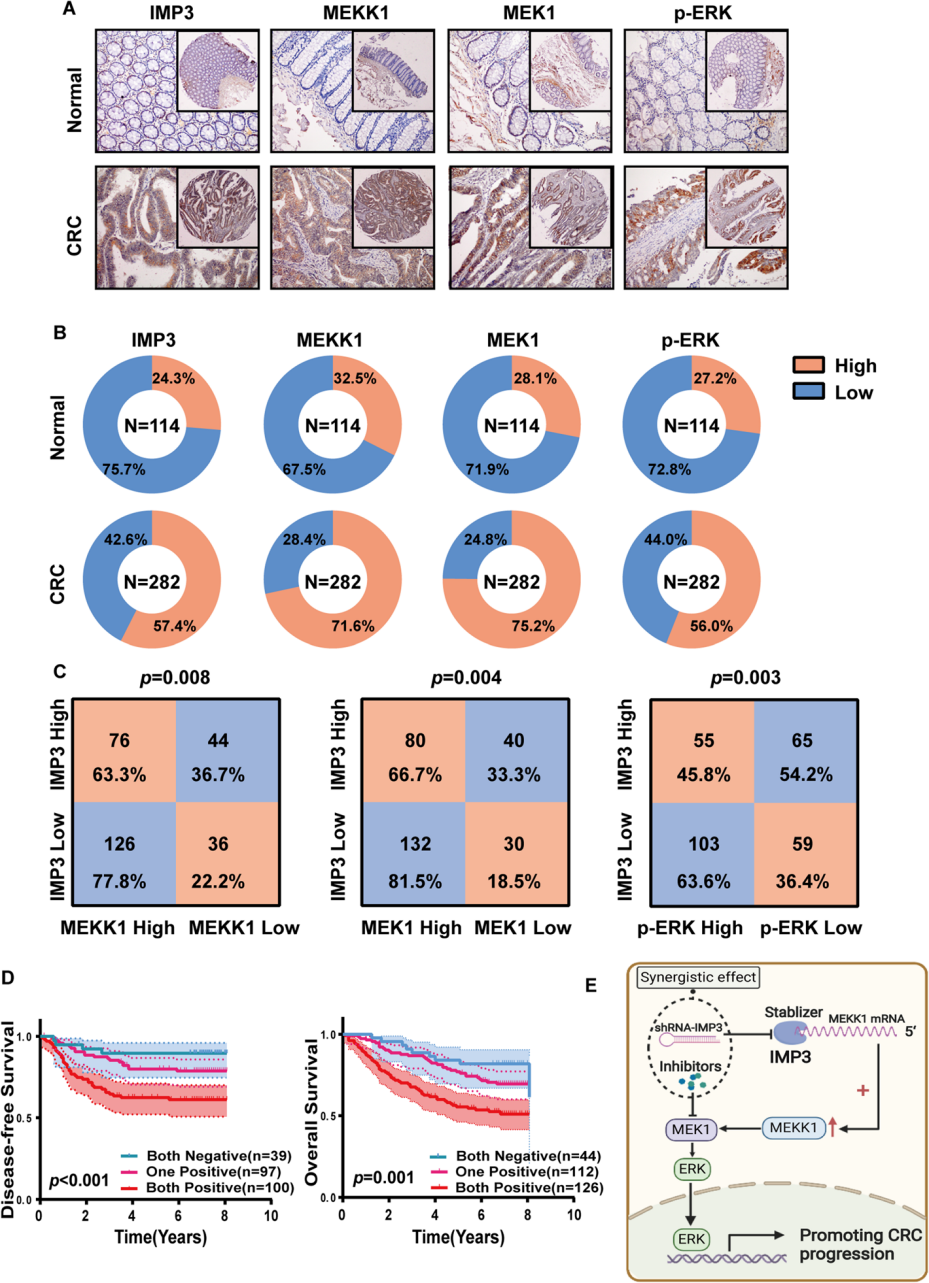
Expression of IMP3 and MEKK1/MEK/ERK in clinical CRC samples. (**A**) Representative images and the number of IMP3, MEKK1, MEK1 and p-ERK detected by IHC in CRC tissue microarrays. (**B**) IMP3, MEKK1, MEK1 and p-ERK expression in adjacent normal colon mucosa (*n* = 114, up panel) and colorectal cancer tissue (*n* = 282, lower panel). (**C**)Correlation of IMP3 expression with MEKK1, MEK1 and p-ERK. (**D**) KaplaneMeier analyses for the FUSCC dataset. Patients were divided into three groups based on the expression of IMP3 and MEKK1 (negative or positive). Both positive groups had the poorest prognosis with the lowest DFS and OS. (**E**)Schematic model showing the role of IMP3 in regulating MEKK1/MEK1/ERK Signaling Pathway in the Progression of Colorectal Cancer

To determine the clinical association between IMP3 and MEKK1, MEK1 and p-ERK in CRC, we assessed the expression of all four of these proteins in the same tissue microarray (*n* = 282). IHC analysis showed that there was a significant difference in the positive expression rate of all the above proteins between CRC tissues and adjacent normal colon mucosa (Fig. 6A, Table 2). MEKK1 was expressed at high levels in 71.6% (202/282) of CRC cancer tissues, 212 (75.2%) patients showed high expression of MEK1, and 158 (56.0%) patients showed high expression of p-ERK (Fig. 6B). The correlations between IMP3, MEKK1, MEK1 and p-ERK with the clinicopathological features of CRC patients are summarized in Table 2. Moreover, consistent with our previous data, expression levels of MEKK1, MEK1 and p-ERK in patients with high IMP3 expression were significantly higher than those in patients with low IMP3 expression (Fig. 6C).

**Table 2:** Correlation between IMP3, MEKK1, MEK1, p-ERK expression and clinicopathologic features in 282 cases of CRC tissues.

Parameters	n	IMP3			MEKK1			MEK1			p-ERK		
		-	+	P value	-	+	P value	-	+	P value	-	+	P value
Age(years)													
<57	133	60	73	0.411	35	98	0.470	34	99	0.785	58	75	0.908
≥57	149	60	89		45	104		36	113		66	83	
Gender													
Male	165	66	99	0.303	50	115	0.392	37	128	0.268	71	94	0.705
Female	117	54	63		30	87		33	84		53	64	
AJCC stage													
I+II	102	54	48	0.008*	37	65	0.027*	28	74	0.442	54	48	0.022*
III+IV	180	66	114		43	137		42	138		70	110	
T stage													
T1+T2	47	27	20	0.024*	17	30	0.194	12	35	0.902	25	22	0.163
T3+T4	235	93	142		63	172		58	177		99	136	
N stage													
N0	117	60	57	0.013*	41	76	0.036*	33	84	0.268	58	59	0.111
N1+N2	165	60	105		39	126		37	128		66	99	
M stage													
Yes	46	14	32	0.069	12	34	0.707	10	36	0.597	17	29	0.295
No	236	106	130		68	168		60	176		107	129	
Differentiation													
Poor	69	33	36	0.308	21	48	0.661	17	52	0.967	31	38	0.854
Well and moderate	213	87	126		59	154		53	160		93	120	

Survival analysis indicated that high expression of MEKK1, MEK1 and p-ERK did not show association with poor OS and DFS (Supplementary Fig. 5A-C). To further determine the combined application of IMP3 and MEKK1 in evaluating the prognosis of CRC patients, CRC patients were divided into three groups based on their IMP3 and MEKK1 expression levels. Patients with positive IMP3 and MEKK1 expression had the poorest OS and DFS. In contrast, those with negative IMP3 and MEKK1 expression had the best OS (*p* < 0.001) and DFS (*p* = 0.001) (Fig. 6D). Collectively, these data identified the importance of the IMP3/MEKK1/MEK/ERK signaling axis in CRC progression (Fig. 6E).

## Discussion

Recent studies have reported a posttranscriptional function for RBPs in the initiation and progression of cancer [19–21]. Further investigations into RBPs in CRC may help identify novel targets to design precise therapeutic treatments [22, 23]. In our study, we first identified the crucial RBP genes by performing RNA-Seq analysis of 8 paired primary colorectal cancers with liver metastases samples, including primary tumor tissues(T), normal adjacent tissues(N) and liver metastatic tissues(M)at the same time. We found that only IMP3 was found to increase from N to T tissue, and increase again from T to M tissue. This result is consistent with the recent study about the proteogenomic analysis of human colon cancer [24], they found that IMP3 was elevated in colon cancer tissue compared with normal colon tissue.

IMP3, a member of RBPs, was found upregulation in several cancers, such as triple negative breast cancer, gastric cancer, pancreatic cancer, and almost all gynecological tumors [25–28]. Moreover, upregulation of IMP3 is predictive of poorer patient survival and a higher probability of distal metastasis [29, 30], suggesting that IMP3 is critical for the progression of human cancer. However, study of the function and the mechanisms of IMP3 in CRC is still limited. In present study, we verified IMP3 was upregulated in CRC using TCGA public database and the cohort from our center, which was positively associated with the AJCC stage, tumor invasion depth, and nodular metastasis; furthermore, patients with higher IMP3 expression had poorer prognoses. To study the pathogenic functions of IMP3, we also generated transgenic mice with a colon-specific deletion of the *IMP3* floxed target allele through the *Villin*-Cre recombinase transgene (*Villin*-Cre). There was a significant reduction in the total tumor area and in the number of colon tumors induced by AOM/DSS treatment in *Villin*-Cre *IMP3*^−/−^ mice when compared to *IMP3*^fl/fl^ mice. In CRC cells, exogenous IMP3 promoted cell proliferation, migration, and invasion both in vitro and in vivo.

It has been shown that IMP3 targets mRNAs for *WNT5B, CD44* and the transcriptional factor *HMGA2* [13, 15, 16]. IMP3 is also required in the context of the *LIN28B* overexpression that drives liver cancer in murine models [31]. LIN28B suppresses expression of Let-7 miRNA, a miRNA which can otherwise inhibit tumor cell migration and invasion by targeting IMP3 [16]; thus, IMP3 may regulate both development and the oncogenic processes of cancer. To search for the molecular targets of IMP3 in CRC, we performed RIP-Seq combined with RNA-Seq analyses and identified 622 direct IMP3 regulatory targets. GO and KEGG pathway analysis showed that these targets were mainly enriched in cancer, RNA binding, and transcription, indicating an important role for IMP3 in cancer. MEKK1, a signaling hub between multiple pathways, was one of the direct IMP3 regulatory targets. We performed RIP assays to verify the binding of IMP3 and MEKK1 mRNA. qRT-PCR and western blot assays showed that IMP3 regulated MEKK1 mRNA and protein levels and downstream activation of the MEK/ERK pathway. GEPIA analysis showed a correlation between IMP3 and MEKK1 expression. Luciferase assays showed that IMP3 could directly bind to the MEKK1 3′-UTR. Overexpression of IMP3 increased the stability of MEKK1 mRNA in the presence of actinomycin D. Knockdown of MEKK1 or the administration of MEK inhibitors abolished the effects of IMP3 on proliferation, migration, and invasion in CRC cells stably overexpressing IMP3.

Mitogen-activated protein kinase (MAPK) signaling cascades are central nodes in a complex signal transduction network that allow eukaryotic cells to respond to a broad set of environmental signals, and all eukaryotic cells possess multiple MAPK pathways [32, 33]. The signal transduction processes of MAPK pathways are typical cascades, the receptor activates the upstream kinase, and causes MAPK members (MAPKKK→MAPKK→MAPK) to be activated sequentially [34]. MEKK1, also known as mitogen-activated protein/ERK kinase kinase 1, is a member of MAPKKK family. MEKK1 regulates three canonical MAPK pathways, including the JNK pathway, the p38 MAPK pathway, and the MEK/ERK pathway [35]. In our study, altered IMP3 expression regulated MEKK1 levels and activation of the MEK/ERK pathway. Studies have reported that MEKK1 may function as a scaffold protein and interact with multiple proteins, playing an important role in cell growth, differentiation, and metastasis in several cancers, such as gastric cancer, pancreatic cancer, and ovarian cancer [36–39]. However, the role of MEKK1 in CRC is still unclear; a series of cell function assays were performed in our study and the results showed that MEKK1 promoted CRC cell proliferation and metastasis.

Despite the administration of epidermal growth factor receptor antibody therapy to CRC patients increasing patient survival, patients with RAS mutations could not be enrolled in neoadjuvant chemotherapy. MEK inhibitors are becoming a promising therapeutic strategy in CRC. Our study showed that U0126, an inhibitor of the MEK/ERK pathway, could reverse IMP3-promoted cell proliferation and metastasis, or had a synergistic effect, in vitro and in vivo; furthermore, this function was independent of RAS mutations, as both RAS mutant and wild-type cell lines were used in these functional assays. These results provide new insights into potential applications for MEK inhibitors in CRC targeted therapy.

## Conclusion

In summary, our study demonstrated that IMP3 has important functions in CRC progression and showed that IMP3 activated the MEK/ERK pathway by directly binding to the MEKK1 3′-UTR, which might reflect the underlying molecular mechanisms of their biological functions. These findings provide a better understanding of the roles of RBPs in CRC progression. Together with further research, these findings may prove to be clinically useful strategies for CRC treatment through inhibiting IMP3 or combing with the MEK1 inhibitor.

## Supplementary Information

**Additional file 1: Supplemental table 1.** Sequence of primers for Quantitative reverse transcription-PCR. **Supplementary Figure 1.** Representative images of wound healing assays and transwell invasion assay for HCT116 and SW480. **Supplementary Figure 2.** The GO analysis by DAVID. **Supplementary Figure 3.** IMP3 promotes CRC progression through the MEKK1/MEK1/ERK pathway. **Supplementary Figure 4.** MEKK1 mimic the function of IMP3 in CRC cells. **Supplementary Figure 5.** The DFS and OS survival curves of MEKK1, MEK1 and p-ERK.

## Data Availability

The datasets used and analyzed during the current study are available from the corresponding author on reasonable request.

## Abbreviations

CRC: colorectal cancer
RBPs: RNA-binding proteins
RNA-seq: RNA-sequencing
IMP3: insulin-like growth factor 2 mRNA-binding protein 3
OS: overall survival
DFS: disease-free survival
FUSCC: Fudan University Shanghai Cancer Center
RIP: RNA immunoprecipitation
GO: Gene Ontology
KEGG: Kyoto Encyclopedia of Genes and Genomes
AOM: azoxymethane
DSS: dextran sodium sulfate

## References

[1] Siegel RL, Miller KD, Fedewa SA, Ahnen DJ, Meester RGS, Barzi A, Jemal A (2017). Colorectal cancer statistics, 2017. CA Cancer J Clin.

[2] Schoumacher M, Hurov KE, Lehar J, Yan-Neale Y, Mishina Y, Sonkin D (2014). Inhibiting Tankyrases sensitizes KRAS-mutant cancer cells to MEK inhibitors via FGFR2 feedback signaling. Cancer Res.

[3] Normanno N, Tejpar S, Morgillo F, De Luca A, Van Cutsem E, Ciardiello F (2009). Implications for KRAS status and EGFR-targeted therapies in metastatic CRC. Nat Rev Clin Oncol.

[4] Xue Z, Vis DJ, Bruna A, Sustic T, van Wageningen S, Batra AS, Rueda OM, Bosdriesz E, Caldas C, Wessels LFA, Bernards R (2018). MAP3K1 and MAP2K4 mutations are associated with sensitivity to MEK inhibitors in multiple cancer models. Cell Res.

[5] Prahallad A, Bernards R (2016). Opportunities and challenges provided by crosstalk between signalling pathways in cancer. Oncogene.

[6] Sugiura R, Kita A, Shimizu Y, Shuntoh H, Sio SO, Kuno T (2003). Share. Feedback regulation of MAPK signalling by an RNA-binding protein. Nature..

[7] Pereira B, Billaud M, Almeida R (2017). RNA-binding proteins in Cancer: old players and new actors. Trends in Cancer.

[8] Gerstberger S, Hafner M, Tuschl T (2014). A census of human RNA-binding proteins. Nat Rev Genet.

[9] Hong S (2017). RNA binding protein as an emerging therapeutic target for Cancer prevention and treatment. Journal of Cancer Prevention.

[10] Bell JL, Wächter K, Mühleck B, Pazaitis N, Köhn M, Lederer M, Hüttelmaier S (2013). Insulin-like growth factor 2 mRNA-binding proteins (IGF2BPs): post-transcriptional drivers of cancer progression?. Cell Mol Life Sci.

[11] Fadare O, Liang SX, Crispens MA, Jones HW, Khabele D, Gwin K (2013). Expression of the oncofetal protein IGF2BP3 in endometrial clear cell carcinoma: assessment of frequency and significance. Hum Pathol.

[12] Degrauwe N, Suva ML, Janiszewska M, Riggi N, Stamenkovic I (2016). IMPs: an RNA-binding protein family that provides a link between stem cell maintenance in normal development and cancer. Genes Dev.

[13] Samanta S, Guru S, Elaimy AL, Amante JJ, Ou J, Yu J, Zhu LJ, Mercurio AM (2018). IMP3 stabilization of WNT5B mRNA facilitates TAZ activation in breast Cancer. Cell Rep.

[14] Glisovic T, Bachorik JL, Yong J, Dreyfuss G (2008). RNA-binding proteins and post-transcriptional gene regulation. FEBS Lett.

[15] Vikesaa J, Hansen TV, Jonson L, Borup R, Wewer UM, Christiansen J, Nielsen FC (2006). RNA-binding IMPs promote cell adhesion and invadopodia formation. EMBO J.

[16] Jonson L, Christiansen J, Hansen TV, Vikesa J, Yamamoto Y, Nielsen FC (2014). IMP3 RNP safe houses prevent miRNA-directed HMGA2 mRNA decay in cancer and development. Cell Rep.

[17] Palanichamy JK, Tran TM, Howard JM, Contreras JR, Fernando TR, Sterne-Weiler T, Katzman S, Toloue M, Yan W, Basso G, Pigazzi M, Sanford JR, Rao DS (2016). RNA-binding protein IGF2BP3 targeting of oncogenic transcripts promotes hematopoietic progenitor proliferation. J Clin Invest.

[18] Zhang M, Weng W, Zhang Q, Wu Y, Ni S, Tan C, Xu M, Sun H, Liu C, Wei P, du X (2018). The lncRNA NEAT1 activates Wnt/beta-catenin signaling and promotes colorectal cancer progression via interacting with DDX5. J Hematol Oncol.

[19] Wang E, Lu SX, Pastore A, Chen X, Imig J, Chun-Wei Lee S, Hockemeyer K, Ghebrechristos YE, Yoshimi A, Inoue D, Ki M, Cho H, Bitner L, Kloetgen A, Lin KT, Uehara T, Owa T, Tibes R, Krainer AR, Abdel-Wahab O, Aifantis I (2019). Targeting an RNA-binding protein network in acute myeloid leukemia. Cancer Cell.

[20] Wang J, Choi JM, Holehouse AS, Lee HO, Zhang X, Jahnel M, Maharana S, Lemaitre R, Pozniakovsky A, Drechsel D, Poser I, Pappu RV, Alberti S, Hyman AA (2018). A molecular grammar governing the driving forces for phase separation of prion-like RNA binding proteins. Cell..

[21] Hodson DJ, Screen M, Turner M (2019). RNA-binding proteins in hematopoiesis and hematological malignancy. Blood..

[22] Kudinov AE, Karanicolas J, Golemis EA, Boumber Y (2017). Musashi RNA-binding proteins as Cancer drivers and novel therapeutic targets. Clin Cancer Res.

[23] Chatterji P, Rustgi AK (2018). RNA binding proteins in intestinal epithelial biology and colorectal Cancer. Trends Mol Med.

[24] Vasaikar S, Huang C, Wang X, Petyuk VA, Savage SR, Wen B, Dou Y, Zhang Y, Shi Z, Arshad OA, Gritsenko MA, Zimmerman LJ, McDermott JE, Clauss TR, Moore RJ, Zhao R, Monroe ME, Wang YT, Chambers MC, Slebos RJC, Lau KS, Mo Q, Ding L, Ellis M, Thiagarajan M, Kinsinger CR, Rodriguez H, Smith RD, Rodland KD, Liebler DC, Liu T, Zhang B, Pandey A, Paulovich A, Hoofnagle A, Mani DR, Chan DW, Ransohoff DF, Fenyo D, Tabb DL, Levine DA, Boja ES, Kuhn E, White FM, Whiteley GA, Zhu H, Zhang H, Shih IM, Bavarva J, Whiteaker J, Ketchum KA, Clauser KR, Ruggles K, Elburn K, Hannick L, Watson M, Oberti M, Mesri M, Sanders ME, Borucki M, Gillette MA, Snyder M, Edwards NJ, Vatanian N, Rudnick PA, McGarvey PB, Mertins P, Townsend RR, Thangudu RR, Rivers RC, Payne SH, Davies SR, Cai S, Stein SE, Carr SA, Skates SJ, Madhavan S, Hiltke T, Chen X, Zhao Y, Wang Y, Zhang Z (2019). Proteogenomic analysis of human Colon Cancer reveals new therapeutic opportunities. Cell..

[25] Samanta S, Sun H, Goel HL, Pursell B, Chang C, Khan A, Greiner DL, Cao S, Lim E, Shultz LD, Mercurio AM (2016). IMP3 promotes stem-like properties in triple-negative breast cancer by regulating SLUG. Oncogene..

[26] Burdelski C, Jakani-Karimi N, Jacobsen F, Möller-Koop C, Minner S, Simon R, Sauter G, Steurer S, Clauditz TS, Wilczak W (2018). IMP3 overexpression occurs in various important cancer types and is linked to aggressive tumor features: a tissue microarray study on 8,877 human cancers and normal tissues. Oncol Rep.

[27] Pasiliao CC, Chang CW, Sutherland BW, Valdez SM, Schaeffer D, Yapp DT (2015). The involvement of insulin-like growth factor 2 binding protein 3 (IMP3) in pancreatic cancer cell migration, invasion, and adhesion. BMC Cancer.

[28] Visser NCM, van der Putten LJM, van Egerschot A, Van de Vijver KK, Santacana M, Bronsert P (2019). Addition of IMP3 to L1CAM for discrimination between low- and high-grade endometrial carcinomas: a European network for individualised treatment of endometrial Cancer collaboration study. Hum Pathol.

[29] Tschirdewahn S, Panic A, Püllen L, Harke NN, Hadaschik B, Riesz P, Horváth A, Szalontai J, Nyirády P, Baba HA, Reis H, Szarvas T (2019). Circulating and tissue IMP3 levels are correlated with poor survival in renal cell carcinoma. Int J Cancer.

[30] Kim HY, Ha Thi HT, Hong S (2018). IMP2 and IMP3 cooperate to promote the metastasis of triple-negative breast cancer through destabilization of progesterone receptor. Cancer Lett.

[31] Nguyen LH, Robinton DA, Seligson MT, Wu L, Li L, Rakheja D, Comerford SA, Ramezani S, Sun X, Parikh MS, Yang EH, Powers JT, Shinoda G, Shah SP, Hammer RE, Daley GQ, Zhu H (2014). Lin28b is sufficient to drive liver cancer and necessary for its maintenance in murine models. Cancer Cell.

[32] Kyriakis JM, Avruch J (2001). Mammalian mitogen-activated protein kinase signal transduction pathways activated by stress and inflammation. Physiol Rev.

[33] Remenyi A, Good MC, Bhattacharyya RP, Lim WA (2005). The role of docking interactions in mediating signaling input, output, and discrimination in the yeast MAPK network. Mol Cell.

[34] Asai T, Tena G, Plotnikova J, Willmann MR, Chiu WL, Gomez-Gomez L, Boller T, Ausubel FM, Sheen J (2002). MAP kinase signalling cascade in Arabidopsis innate immunity. Nature..

[35] Chang L, Karin M (2001). Mammalian MAP kinase signalling cascades. Nature..

[36] Luettich K, Schmidt C (2003). TGFbeta1 activates c-Jun and Erk1 via alphaVbeta6 integrin. Mol Cancer.

[37] Bian D, Su S, Mahanivong C, Cheng RK, Han Q, Pan ZK, Sun P, Huang S (2004). Lysophosphatidic acid stimulates ovarian Cancer cell migration via a Ras-MEK kinase 1 pathway. Cancer Res.

[38] Cuevas BD, Winter-Vann AM, Johnson NL, Johnson GL (2006). MEKK1 controls matrix degradation and tumor cell dissemination during metastasis of polyoma middle-T driven mammary cancer. Oncogene..

[39] Su F, Li H, Yan C, Jia B, Zhang Y, Chen X (2009). Depleting MEKK1 expression inhibits the ability of invasion and migration of human pancreatic cancer cells. J Cancer Res Clin Oncol.

