# Supplementary material for: RNA-binding protein IMP3 is a novel regulator of MEK1/ERK signaling pathway in the progression of colorectal Cancer through the stabilization of MEKK1 mRNA
Source: J Exp Clin Cancer Res. 2021 Jun 21;40:200. doi: 10.1186/s13046-021-01994-8 (PMC8215736; doi:10.1186/s13046-021-01994-8)
Supplement: Supplementary file 1 — Additional file 1: Supplemental table 1. Sequence of primers for Quantitative reverse transcription-PCR. Supplementary Figure 1. Representative images of wound healing assays and transwell invasion assay for HCT116 and SW480. Supplementary Figure 2. The GO analysis by DAVID. Supplementary Figure 3. IMP3 promotes CRC progression through the MEKK1/MEK1/ERK pathway. Supplementary Figure 4. MEKK1 mimic the function of IMP3 in CRC cells. Supplementary Figure 5. The DFS and OS survival curves of MEKK1, MEK1 and p-ERK. [file 13046_2021_1994_MOESM1_ESM.docx]

| **Supplemental table1. Sequence of primers for Quantitative reverse transcription-PCR** | | |
| --- | --- | --- |
| Gene | Forward primer (5’------3’) | Reverse primer(5’------3’) |
| IMP3 | ATGACTCCTCCCTACCCG | GAAAGCTGCTTGATGTGC |
| MEKK1 | ACAGCACTGGTCAGAGACTAAGAATTG | ATAGCACAGCCAACACTCCATACATC |
| GAPDH | AGAAGGCTTGGGCTCATTTG | AGGGGCCATCGACAGTCTTC |

**
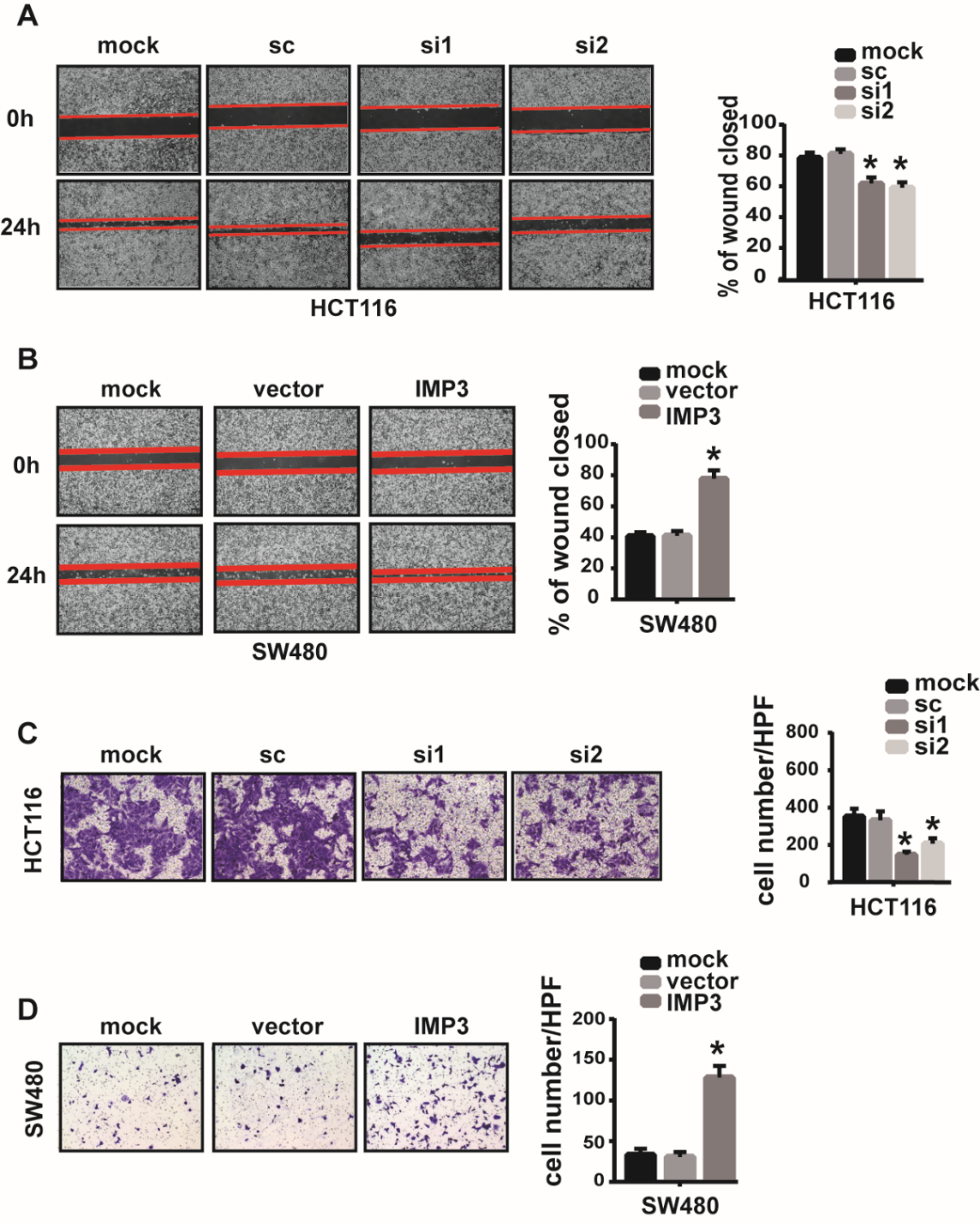
**

**Supplementary Figure1.** Representative images (40×) of wound healing assays for HCT116 (A) and SW480 (B, * p<0.05). Representative images (200×) of transwell invasion assay for HCT116 (C) and SW480 (D, * p<0.05).

**
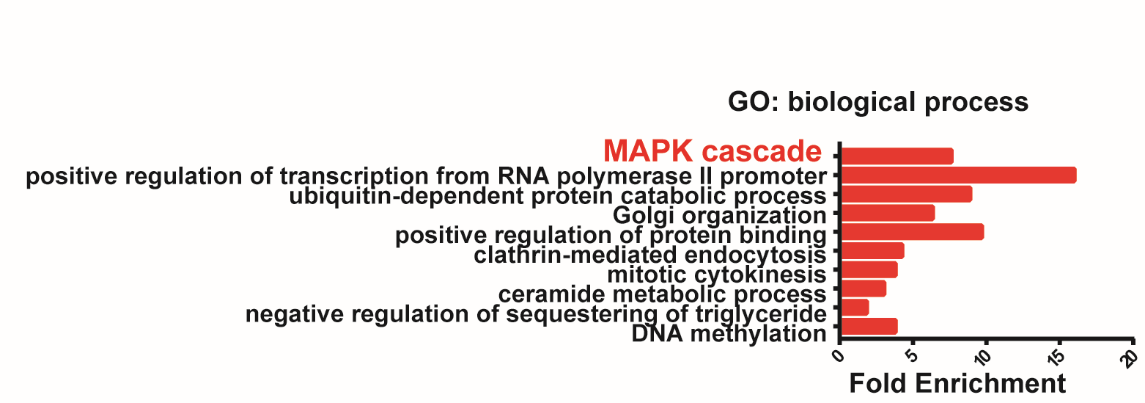
**

**Supplementary Figure2.** The GO analysis by DAVID.

**
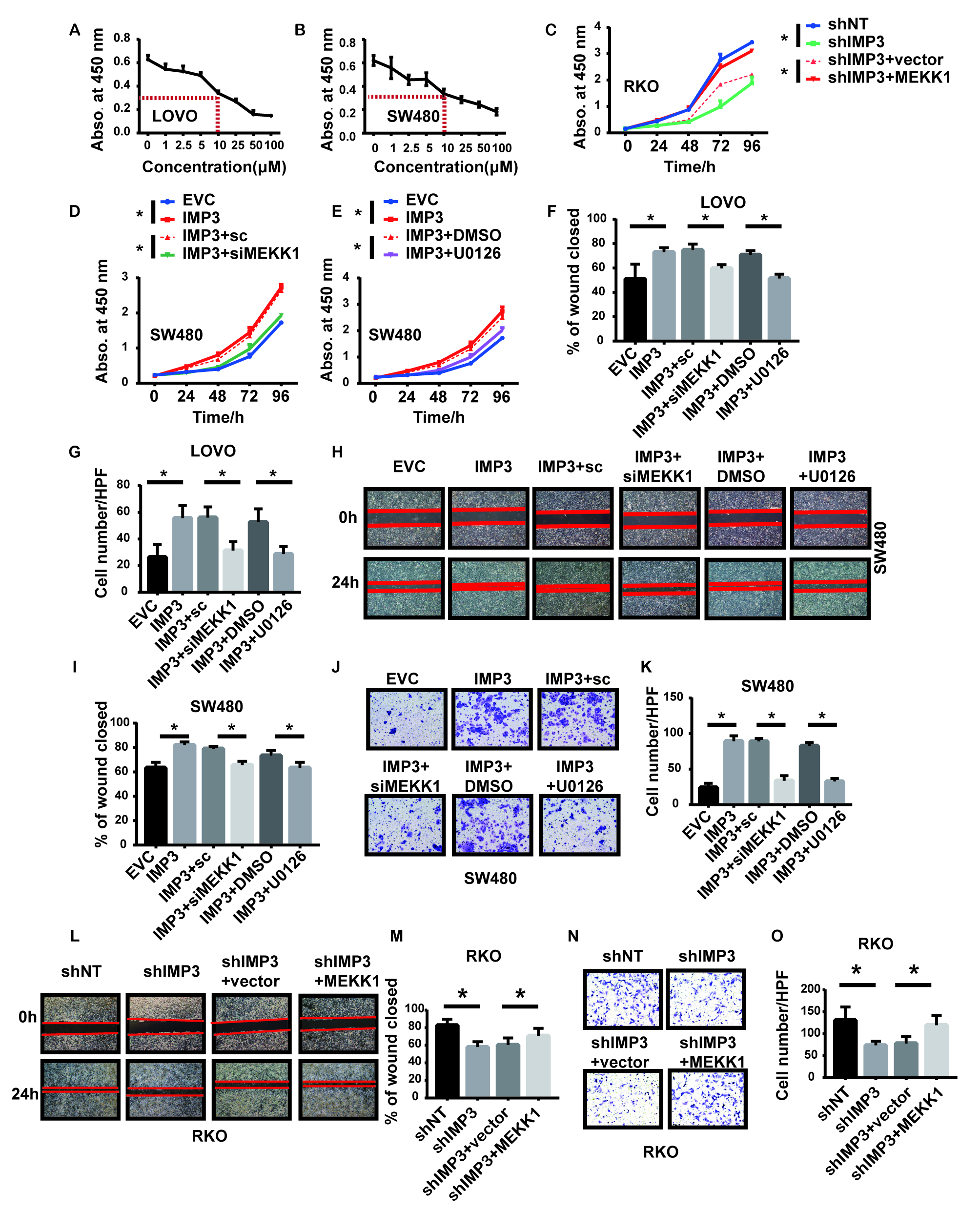
**

**Supplementary Figure3.** The detection of the IC50 of U0126 in LOVO (A) and SW480 (B). (C) CCK-8 results showed that overexpression of MEKK1 reversed the cell viability reduced by knockdown of IMP3 in RKO cells (* *p*<0.05). (D) CCK-8 results showed that knockdown of MEKK1 attenuated the enhanced cell viability induced by overexpression of IMP3 in SW480 cells (* *p*<0.05). (E) CCK-8 results showed that MEK/ERK inhibitor U0126 attenuated the enhanced cell viability induced by overexpression of IMP3 in SW480 cells (* *p*<0.05). (F) The histogram of (F) wound healing assays and (G) transwell assays showed that MEKK1 repression or U0126 rescued the enhanced invasion and migration ability by overexpression of IMP3 (* *p*<0.05). Representative images (H) and histogram (I) of wound healing assays showed that MEKK1 repression or U0126 rescued the enhanced invasion and migration ability by overexpression of IMP3 (* *p*<0.05). Representative images (J) and histogram (K) of transwell assays showed that MEKK1 repression or U0126 rescued the enhanced invasion and migration ability by overexpression of IMP3 (* *p*<0.05). Representative images (L) and histogram (M) of wound healing assays showed that MEKK1 overexpression reversed the invasion and migration ability weakened by knockdown of IMP3 (* *p*<0.05). Representative images (N) and histogram (O) of transwell assays showed that MEKK1 overexpression reversed the invasion and migration ability weakened by knockdown of IMP3 (* *p*<0.05).

**
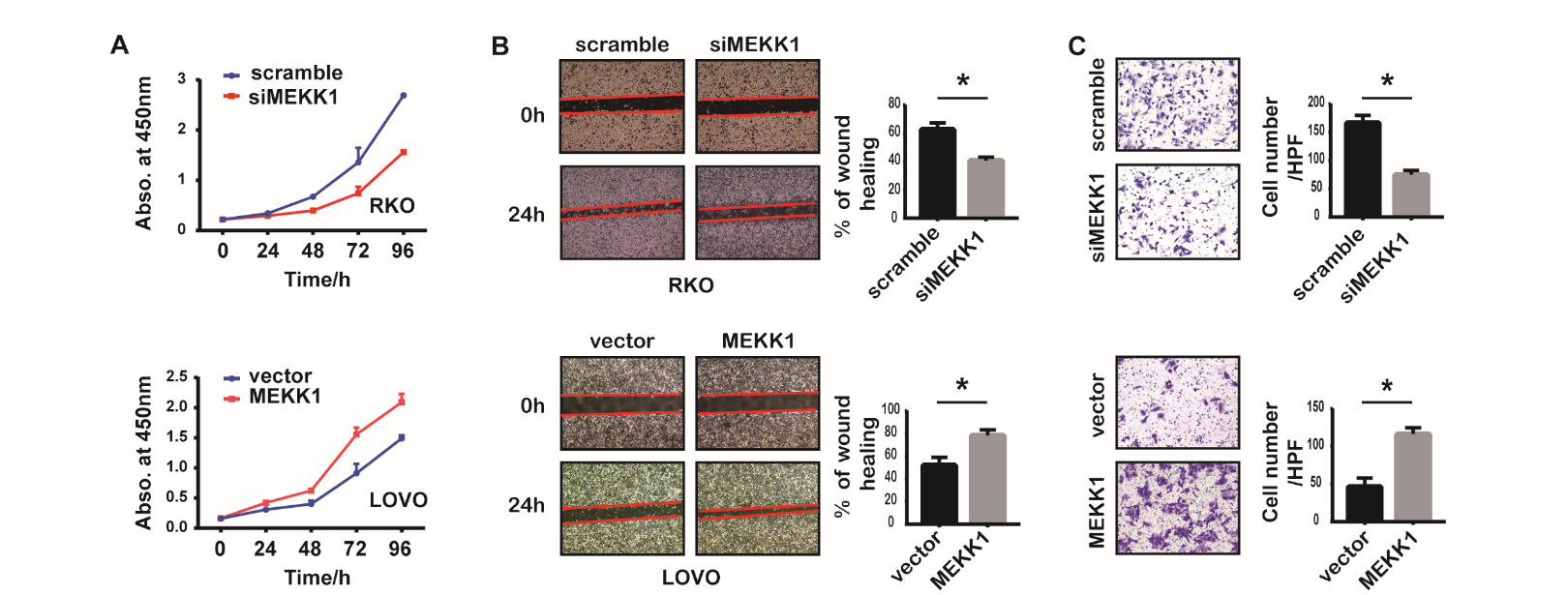
**

**Supplementary Figure4. MEKK1 mimic the function of IMP3 in CRC cells.** (A) CCK8 assays showed that knockdown of MEKK1 reduced cell viability in RKO cells, and upregulation of MEKK1 increased cell viability in LOVO cells (* *p*<0.05). (B) Representative images (40×) of wound healing assays for indicated cells (* p<0.05). (C) Representative images (200×) of transwell invasion assay for indicated cells (* p<0.05).

**
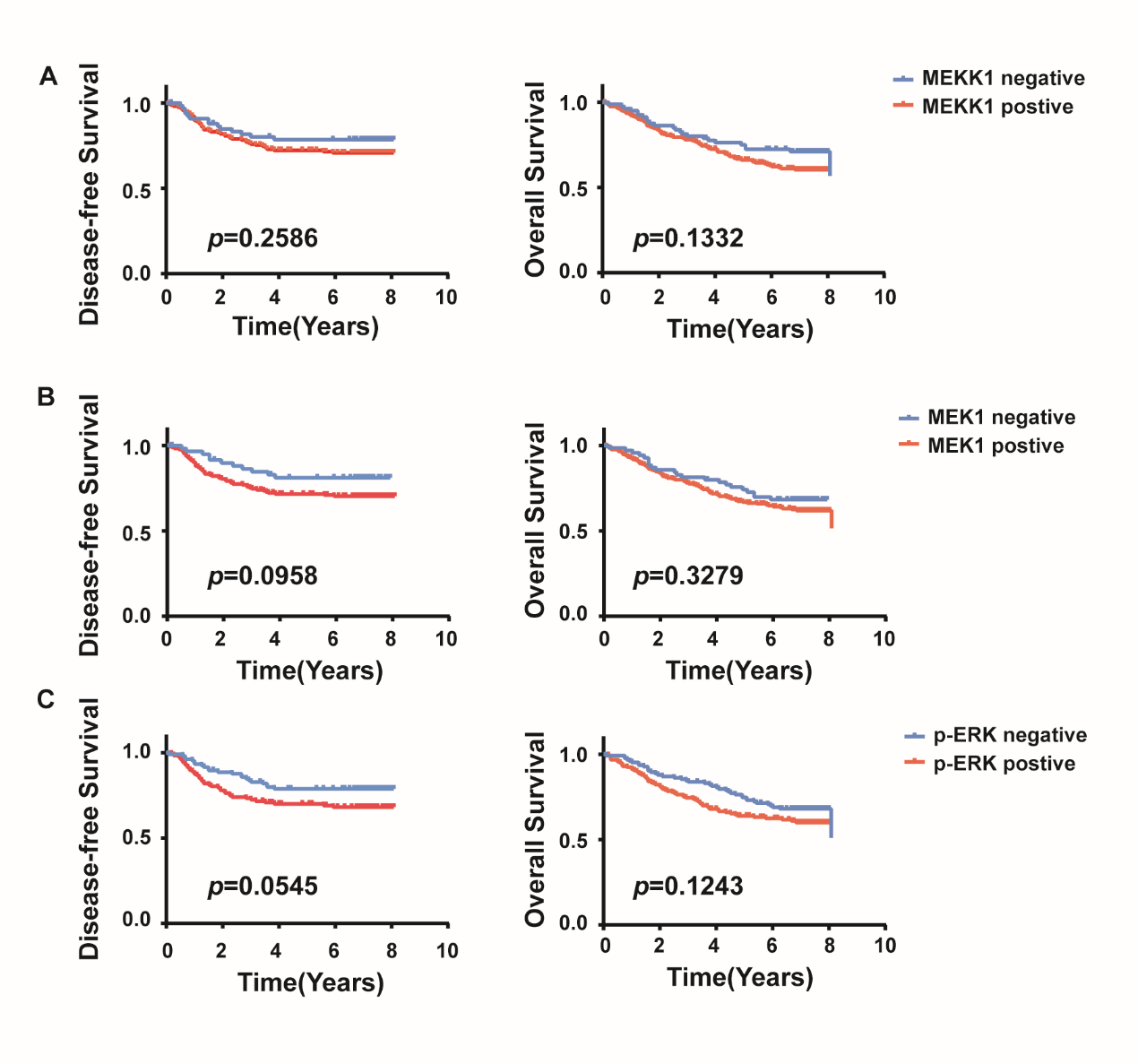
**

**Supplementary Figure5.** The DFS and OS survival curves of MEKK1 (A), MEK1 (B) and p-ERK (C) by Kaplane-Meier analyses for the FUSCC dataset.
